# Pelagic metagenome-assembled genomes from an estuarine salinity gradient in San Francisco Bay

**DOI:** 10.1128/MRA.00800-23

**Published:** 2023-11-06

**Authors:** Anna N. Rasmussen, Christopher A. Francis

**Affiliations:** 1 Department of Earth System Science, Stanford University, Stanford, California, USA; DOE Joint Genome Institute, Berkeley, California, USA

**Keywords:** estuary, metagenome-assembled genomes, pelagic, gradients

## Abstract

San Francisco Bay (SFB) is a large and highly human-impacted estuarine system. We produced 449 metagenome-assembled genomes from SFB waters, collected along the salinity gradient, providing a rich data set to compare the metabolic potential of microorganisms from different salinity zones within SFB and to other estuarine systems.

## ANNOUNCEMENT

Despite the ecological, cultural, and economic importance of San Francisco Bay (SFB), few studies have published microbial metagenome-assembled genomes (MAGs) from SFB (1, 2). We submitted eight bottom water samples from along the salinity gradient for metagenomic sequencing particularly to gain insights into nitrifier ecology in ammonia-rich SFB waters. Using a variety of methods, we created a library of representative MAGs from riverine to brackish to marine waters of SFB. We previously reported on ammonia-oxidizing archaea MAGs, including a massive bloom lineage captured by this data set (1), and the ecogenomics of *Pelagibacterales* (SAR11) subclade IIIa (2); however, most of the rich genomic data we generated remain to be explored.

Sample collection, DNA extraction, sequencing, and assembly methods were previously described (1). Briefly, 400–1,000 mL of bottom water (1 m above estuary floor) was collected from the SFB channel on 24th and 25th October 2013, at eight stations during United States Geological Survey (USGS) Water Quality monitoring cruises, including stations 657 (fresh, <0.5 PSU), 649 (oligohaline, 0.5–5 PSU), 3 (mesohaline, 5–18 PSU), 9 and 13 (polyhaline, 18–30 PSU), and 18, 27, and 34 (euhaline, 30–40 PSU). Microbial biomass was collected on 0.22-µm filters after passage through a 10-µm pore size pre-filter, and DNA was extracted using the FastDNA SPIN Kit for Soil (MP Biomedicals, Santa Ana, CA). Library preparation and metagenomic sequencing were performed by the DOE Joint Genome Institute (JGI) via a CSP project (Proposal ID 503022) to create Illumina fragments (300 bp) sequenced on an Illumina HiSeq 2500-1TB generating paired 150 bp length reads. For library preparation, 100 ng of DNA was sheared (to 479–610 bp) using the Covaris LE220 and size was selected using SPRI beads (Beckman Coulter). The fragments were treated with end-repair, A-tailing, and ligation of Illumina-compatible adapters (IDT, Inc.) using the KAPA-Illumina library creation kit (Kapa Biosystems). qPCR was used to determine the concentration of the libraries. We used quality-controlled and filtered metagenome data generated by JGI using their standard BBtools (v38.87) (3) pipeline (v3.7.3) for assembly, binning, and refining using the metaWRAP (v1.3.2) pipeline (4). Additionally, metagenomes were subset using Seqtk (https://github.com/lh3/seqtk) to 5%, 10%, 20%, and 50% of reads and co-assembled based on salinity zone. All metagenomes, subsets, and co-assemblies were assembled and binned as previously described (1) using metaSPAdes (v3.13.0) (5), MEGAHIT (v1.1.3) (6), MaxBin 2.0 (v2.2.6) (7), and MetaBAT2 (v2.12.1) (8). Bins were consolidated and filtered using metawrap bin_refinement to have >50% completeness and <10% contamination and then reassembled with metaSPAdes. MAGs were dereplicated using dRep (v2.3.2) (9) at 95% average nucleotide identity (ANI) and then taxonomically classified using the Genome Taxonomy Database toolkit (GTDB-tk v2.1.1; GTDB R07-RS207) (10).

**Fig 1:**
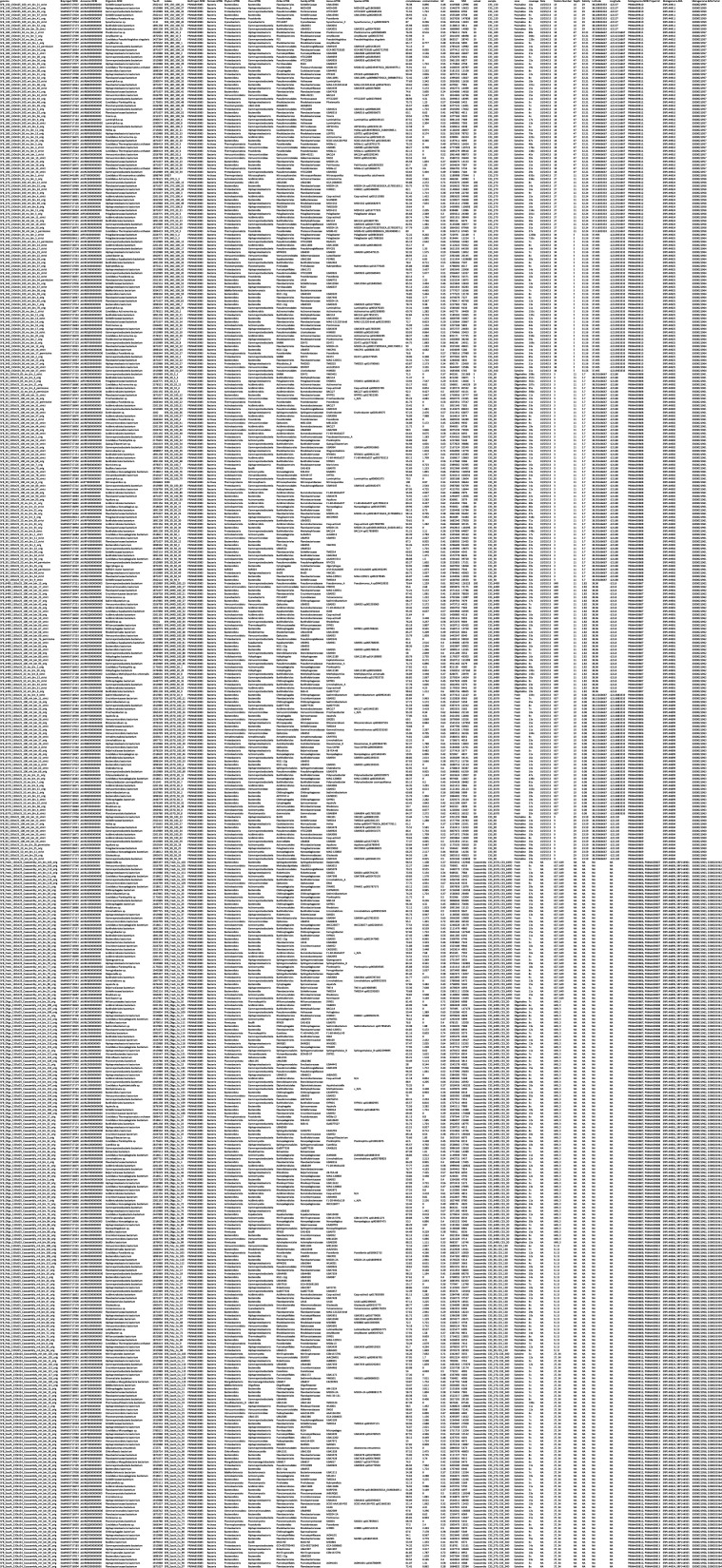
Accession numbers, assembly statistics, taxonomic assignments, and sample data for MAGs also available at https://doi.org/10.6084/m9.figshare.24085767.

**Fig 2:**
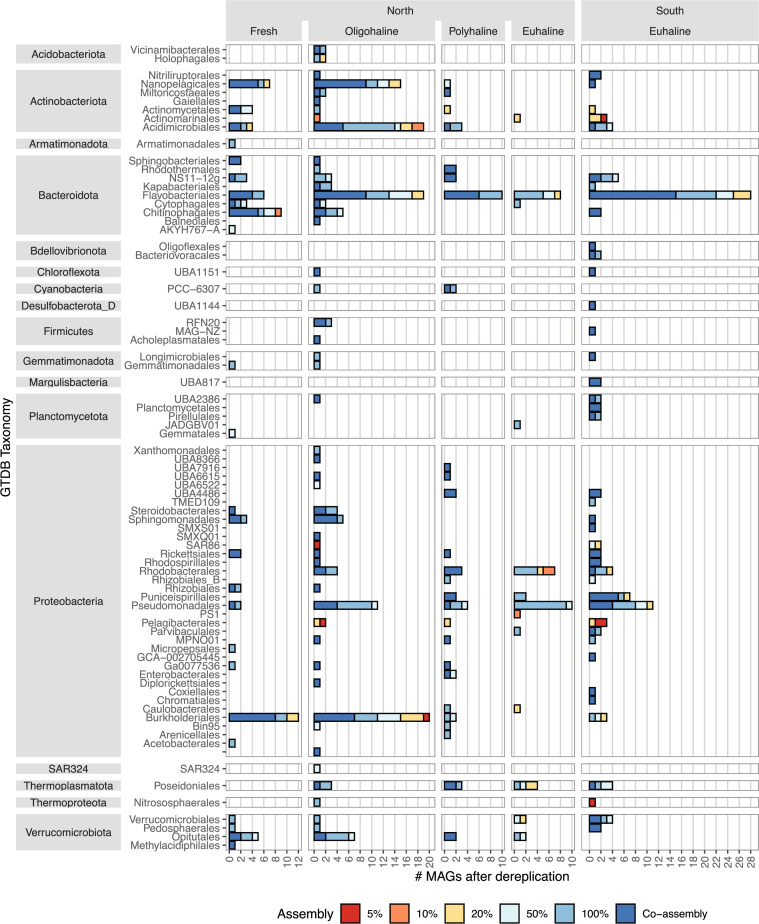
Number of MAGs (*x*-axis) in data set by GTDB-tk classification (order grouped by phylum) on the *y*-axis. Bar color indicates the metagenome treatment from which a representative MAG was binned (either subset, all reads [100%], or co-assembly). Plot faceted by salinity zone and by Bay region (North and South).

Here, we present a total of 449 MAGs representing 17 phyla, including many MAGs from *Proteobacteria* (*n* = 178), *Bacteroidota* (*n* = 118), and *Actinobacteriota* (*n* = 73) (Fig. 1). Binning from subset metagenomes yielded MAGs from high-coverage genera such as *Pelagibacter*, *Nitrosopumilus*, or TMED189 (*Actinobacteria)* (Fig. 2). A median of 10.6× (range 4× to 103×) genome coverage was optimal for recovering genomes. An average of 24% (range 19%–28%) of metagenomic reads were competitively recruited back to the dereplicated MAG library using Bowtie2 (v2.4.2) (11) with the default minimum score threshold. This data set allows for comparisons of closely related microorganisms from different salinity zones and increases our understanding of estuarine pelagic microbial communities. These samples have corresponding 16S rRNA amplicon libraries (12).

## Data Availability

Raw metagenomes are available under NCBI BioProject no. PRJNA439806 to PRJNA439813 (NCBI SRA accession numbers: SRR7130817, SRR7130819, SRR7130820, SRR7130903, SRR7131305, SRR7131306, SRR7132116, SRR7132117). Quality controlled and filtered metagenomes are available from the JGI Genome Portal under accession numbers 3300021957 to 3300021964. MAG assemblies are deposited in NCBI BioProject no. PRJNA819083, as are links to BioSamples for the two ammonia-oxidizing archaea MAGs (assemblies are stored under PRJNA439808 and PRJNA439812) (Fig. 1, column Accession.NCBI). Corresponding 16S rRNA amplicon libraries are available in NCBI BioProject no. PRJNA577706. Figure 1 contains relevant MAG metadata (e.g., quality, taxonomy, accession numbers, etc.).
